# Low utilization of postnatal care: searching the window of opportunity to save mothers and newborns lives in Islamabad capital territory, Pakistan

**DOI:** 10.1186/s13104-015-1646-2

**Published:** 2015-11-04

**Authors:** Nighat Sultana, Babar Tasneem Shaikh

**Affiliations:** Department of Health Systems and Policy, Health Services Academy, Park Road, Chak Shahzad, Islamabad, 44000 Pakistan

**Keywords:** Postnatal care, Reproductive health services, Maternal, Newborn and child health, Pakistan

## Abstract

**Background:**

Each year an estimated 289,000 maternal deaths occur worldwide; of which 50 % of maternal deaths occur around labor, delivery and immediate postpartum period. Postnatal care (PNC) is crucial but relatively neglected component in the continuum of care, and hence a missing link toward safe motherhood. Despite the significant improvement in child health and safe motherhood interventions, maternal mortality ratio, perinatal mortality and neonatal mortality still remain a challenge in achieving the Millennium Development Goals 4 and 5 in Pakistan.

**Methods:**

This was a descriptive cross sectional study carried out in the four union councils of Islamabad capital territory, to understand the determinants of postnatal health care seeking. The study sample comprised 225 postpartum women with a child 0 to 1 month of age, and the health care providers from both public and private sector. A pre-tested semi-structured questionnaire was used for data collection. One focus group discussion with health care providers was conducted, using open ended questions guide.

**Results:**

Only 30 % women received PNC; amongst which 68 % went to a government health facility. According to the health service providers, 90 % women are not interested in PNC, and that is because they lack awareness, face mobility and transportation issues, and cannot afford the cost of health care. Besides many other determinants, women’s education was significantly associated with the PNC utilization.

**Conclusion:**

More robust and culturally sensitive campaign on importance of PNC must be thought out by the national MNCH program to inform the less literate and peri urban inhabitants of Islamabad. Health providers ought to be sensitized and trained for promoting PNC to save maternal and newborn lives.

## Background

Many countries still have a high maternal mortality ratio (300–499 deaths per 100,000 live births), and the timing of maternal deaths is clustered around labor, delivery and the immediate postpartum period, extremely high on the first and 2nd day after birth [1]. The postnatal care (PNC) is essential for monitoring complications arising from delivery such as postpartum hemorrhage, puerperal sepsis, fever and infections. Moreover, every year an estimated four million babies die in the first 4 weeks of life; the highest risk being on the first day of life [2]. As defined by WHO (2009), the standard time of early neonatal care is within 24 h of childbirth. Essential newborn care must include hygiene during delivery, including cord care; keeping the newborn warm; early initiation of breastfeeding and exclusive breastfeeding; immunization; care of eyes; care during illness; and care of low birth weight newborns [3]. There is ample evidence that a large proportion of maternal and neonatal deaths can be averted during the first 48 h after delivery with a simple examination. PNC visits provide an ideal opportunity to educate a new mother on how to care for herself and her newborn baby in the first 42 days. That is why the PNC is crucial, yet a relatively less sought component in the continuum of care for mother and the newborn [4].

Despite a significant number of child health and safe motherhood interventions in Pakistan, both by the government and the non-government organizations, maternal mortality ratio, perinatal mortality and neonatal mortality still remain unacceptably high, and the indicators lag far behind the targets of the Millennium Development Goals (MDGs) 4 and 5 [1]. Ironically, not much visible efforts are seen to advocate and promote the PNC, the least utilized component of the maternity services. According to the Pakistan Demographic & Health Survey 2012–2013 report, about 73 % of women are seeking antenatal care (ANC), 48 % of deliveries are conducted at a health facility. Around 60 % of women received PNC for their last birth within the first two days following delivery. However, from second day onward, the percentage of women seeking PNC declines significantly. Among women who had postnatal checkups, 54 % received PNC within 4 h of delivery, 5 % came within 4–23 h, and 2 % sought it after 1–2 days. The rest never had any PNC checkup; and believe that it is not necessary to seek PNC [4].

Our study endeavors to understand the phenomenon of low utilization of PNC among the recently delivered mothers with a child 0 to 1 month of age. This study has also documented the views of the health care providers from both public and private sector to unfold the same conundrum; and for the purpose of developing some practical recommendations to improve the state of affairs.

## Methods

It was a descriptive cross sectional study conducted at four union councils of Islamabad capital territory and included Tumair, Phulgran, Chirrah and Kirpha. Research protocol was approved by the Ethics Review Committee of the Health Services Academy, Islamabad. A two stage sampling technique was used; first the union councils were randomly selected, and then the households were purposively selected, with the assistance of a local lady health worker, who exactly knew in which house a woman has recently delivered. The study sample comprised 225 married women of age 15–45 years who delivered in last 1 month, and health care providers from both public and private sector.

A semi-structured pre-tested questionnaire was used to collect data after informed verbal/written consent from the respondents. The completed questionnaire were checked for errors, edited, cleaned, coded and data was entered into SPSS 16.0 for statistical analysis. Cross-tabulation was carried out between independent variables (age, education, employment status, household income, family size, transport, ANC Visit, delivery place) and dependent variable i.e., PNC. Chi squared test was used as a test of significance.

A focus group discussion was conducted with the health care providers (2 doctors, 2 nurses, 3 lady health workers), using open ended questions guide. Their views and perceptions on non-use of PNC were recorded and triangulated with reasons given by the women respondents.

## Results

### Socio demographic details

Within an age range of 15–45 years, the majority of the women interviewed (69 %) were between 20 and 29 years; 32 % were illiterate; mostly housewives (88 %), with a few on a daily-wage work, agricultural labor or had their own small business. Average monthly income of the household of the respondents was reported PkRs15,000 (USD150 approx.) per month, supporting an average household of size of seven, with a mixed trend in family structure, as shown in Table 1.

**Table 1:** Socio demographic profile of respondents (n = 225)

Age (years)	%
20–29	69
30–39	30
40–45	1
Education	
Illiterate	32
Up to secondary	38
Higher secondary and above	31
Employment status	
Yes	12
No	88
Household income	
<Rs 10,000	35
Rs 10,000–15,000	33
>Rs 15,000	32
Family structure	
Nuclear	50
Extended	50
Years of marriage	
0–5	49
6–10	35
>10	16

### Physical accessibility of the health facility

For distance to the nearest health facility, 34 % women reported to be living within 0–2 km of distance, 30 % women living within 3–5 km, 16 % living within 6–10 km, and 20 % of the women were living at a distance of more than 10 km. With regard to the travelling time to the nearest health facility, the median time reported is 30 min (IQR: 15–60 min). On the availability of the transportation, 84 % of the women confirmed its availability to go to the health facility.

### Women autonomy (social mobility and decision making power)

Women autonomy about social mobility and decision making power were assessed, and several aspects such household expenditure, children’s education, household purchases, decision to consult a health provider were recorded (as shown in Table 2).

**Table 2:** Women in decision making

Indicators	Woman alone (%)	Husband and wife (%)	Only husband (%)	Family members (%)
Decision regarding household expenditures	3	58	16	23
Decision regarding children education	3	69	13	15
Decision to consult health care provider	4	60	11	24

Moreover, we found that 72 % women are allowed to visit health facility but accompanied; only 24 % allowed visiting alone, and 4 % are not allowed to visit health facility at all.

### Health seeking, delivery and postnatal checkup place

**Fig. 1:**
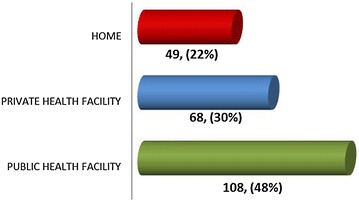
Women’s preferred place of delivery

The government health facilities are significantly preferred and used by 62 % of respondents, while remaining 38 % respondents liked to consult private health facilities. With regard to the delivery, almost half of the women interviewed had the history of delivery at public sector health facility (Fig. 1).

Likewise, preferred place for the postnatal checkup would be a public sector health facility (68 %), followed by 28 % going to a private clinic, and 4 % preferred home for having a PNC consultation.

### Antenatal versus postnatal care

**Fig. 2:**
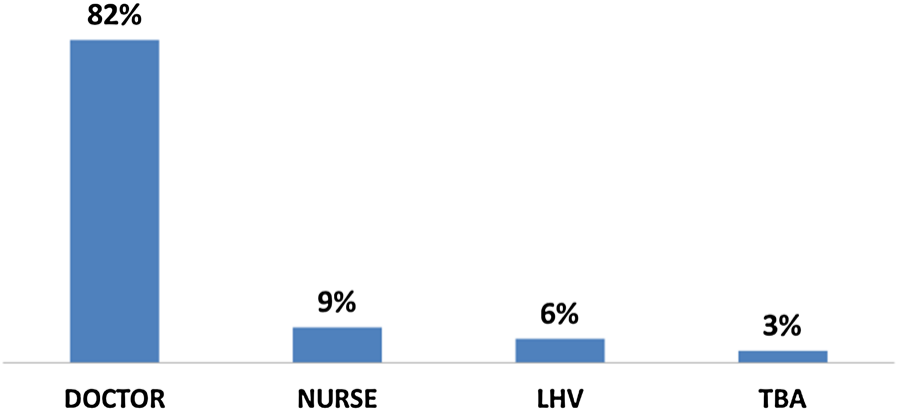
Health provider consulted for PNC

Around 96 % women had received ANC visit, out of this 91 % consulted with skilled healthcare providers (doctors/nurses/midwife/LHV); while remaining 9 % consulted the traditional birth attendants. Only 30 % respondent women consulted for PNC. Out of this, a large majority (82 %) consulted a doctor, while the rest went to a paramedic or a traditional birth attendant (Fig. 2).

**Fig. 3:**
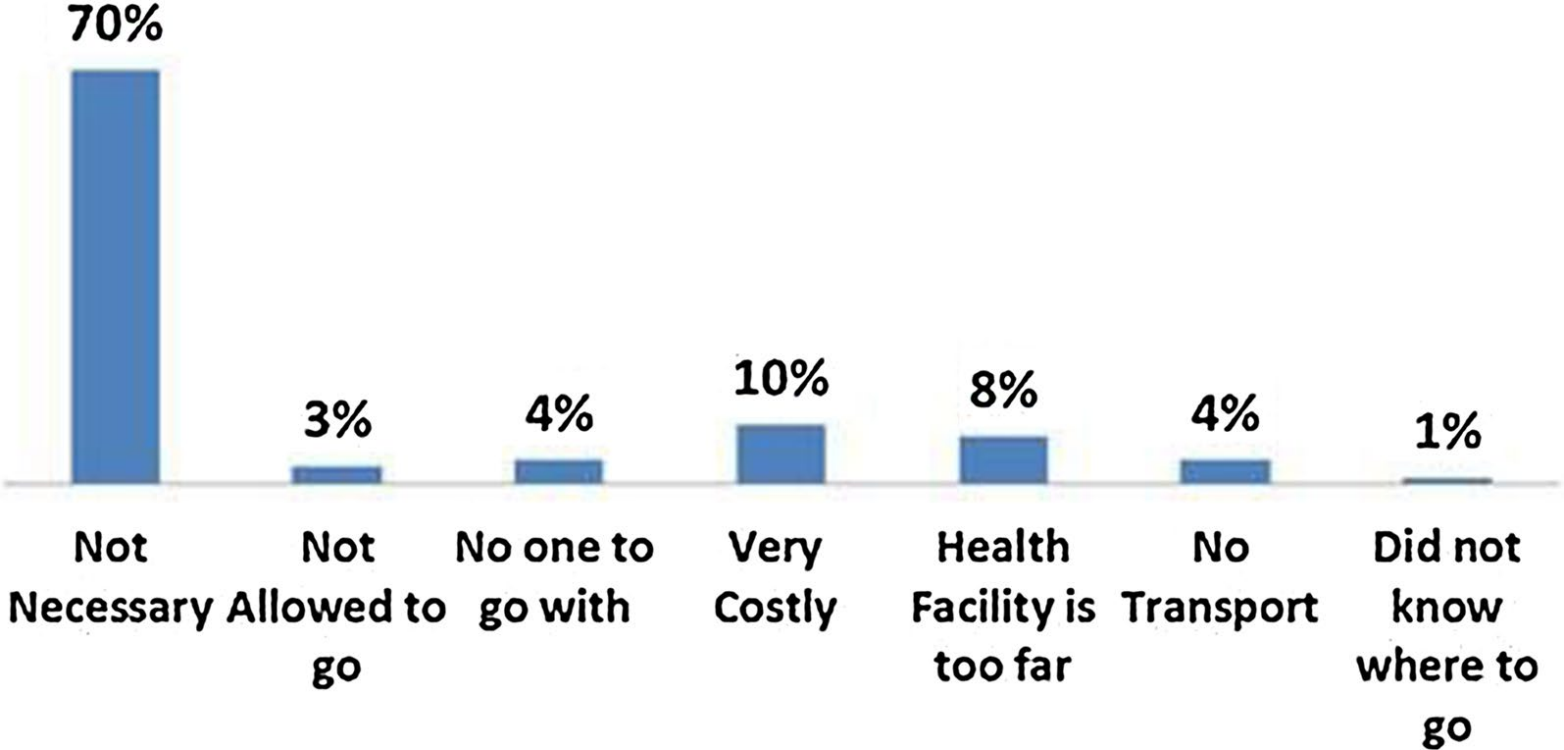
Reasons for not seeking PNC

A large proportion of women (70 %) did not consult with anyone for PNC; out of which a majority (70 %) mentioned that the postnatal checkup was not necessary. Others reasons included affordability, distance of health facility, nobody to accompany etc. (Fig. 3).

When test of significance was applied (Chi square) to see the association of any independent variable with the outcome variable i.e., seeking PNC, it was observed that age of the woman, years of marriage, family structure (joint or nuclear), number of children, household income, and occupation of the woman were not found to have any significant relation with the PNC seeking. However, the education of the women, availability of transport and the delivery assisted by a doctor did show significant association (p value <0.05) with the PNC seeking among women.

As per the health service providers (doctors, nurses, lady health workers), for women not seeking PNC, there are three major reasons: (1) not considered necessary, (2) non-availability of transport, and (3) financial constraint. According to the opinion of service providers, as much as 90 % of women are not interested in PNC due to lack of awareness about its importance. They suggested employing a comprehensive and culturally sensitive reproductive health strategy, for promoting messages related to importance of PNC for mothers as well as the newborn.

## Discussion

Health education and health promotion seem to be a missing or weak link in public health programs of Pakistan, especially focusing on PNC seeking behaviors among women belonging to less literate and low socio-economic stratum. Low PNC utilization in surroundings of Islamabad-the capital territory, is even more worrisome. Nevertheless, social arrangements should be thoughtfully considered to make the health system more responsive [5]. The PNC is least sought consultation, and that is primarily due to lack of awareness among the women, and community at large, about its importance. PNC is critical not only for woman’s own health and postpartum checkup, but it is extremely useful to seek advice for newborn’s issues such as cord care, breast feeding, skin hygiene, immunization etc. [3]. Promoting PNC may potentially save many perinatal and early neonatal deaths. Local media and community based organizations also have a role to play.

The national maternal newborn and child health program must emphasize on messages informing new and expecting mothers about the benefits of PNC for their own and child’s health. Simultaneously, capacity building of health care providers for their own awareness about the postnatal utilization and communication with mothers to create demand of PNC.

Our study corroborates with the fact sheet of WHO which states that unfortunately, the majority of mothers and newborns in low- and middle-income countries do not receive optimal care during these periods [6]. In our study, only 30 % women received PNC, which is a reflection of the overall PNC trend existing in country and in the region [7].The other contributing factors are non-availability of transport, type of service provider, delivery place and education are important factors linked with the utilization of PNC [8]. A study conducted in the rural areas of Nepal, found strong association between ANC and PNC. The main reason of increased uptake of PNC was counseling session and health education given to mothers during the ANC [9, 10]. Therefore, it can be established that during the ANC visits, health care providers must start counseling for the PNC too. Geographical accessibility significantly affects the extent of utilization of PNC services [11, 12]. It is, therefore, imperative to train community health workers to provide PNC to the women at their homes. In this regard, the lady health workers of Pakistan certainly have the potential to redress the problem [13]. The findings of our study also revealed that the likelihood of receiving PNC in literate women is significantly higher as compare to illiterate women, which again is in concordance with other studies showing that women from communities with higher level of education were more likely to receive PNC [14, 15]. Interventions to increase the use of postnatal services should target the uneducated, and those women who live in disadvantaged communities [16, 17].

The study could not be extended to other areas of Islamabad due to financial and time constraints, so generalizability remains limited. Nevertheless, the findings of the study area could be associated with the level of education, demographic and the socio-cultural context which of course is different in other parts of the country.

## Conclusion

In Pakistan, PNC trends have always been low as compared to ANC seeking. Promoting the benefits of PNC will definitely bear fruits and will help in saving many newborn lives and will protect many mothers from postpartum complications. National campaign must focus on PNC messages also while advocating for other components of continuum of care during pregnancy. Health care providers also have a definite role too in creating demand for PNC, and later the responsibility of providing the same with utmost care and empathy.
